# Cardiovascular Risk Factors and Total Serum Antioxidant Capacity in Healthy Men and in Men with Coronary Heart Disease

**DOI:** 10.1155/2014/216964

**Published:** 2014-08-11

**Authors:** Anna Gawron-Skarbek, Jacek Chrzczanowicz, Joanna Kostka, Dariusz Nowak, Wojciech Drygas, Anna Jegier, Tomasz Kostka

**Affiliations:** ^1^Department of Geriatrics, Medical University of Lodz, Pl. Hallera 1, 90-647 Lodz, Poland; ^2^Department of Preventive Medicine, Medical University of Lodz, Zeligowskiego Street 7/9, 90-752 Lodz, Poland; ^3^Department of Hygiene and Health Promotion, Medical University of Lodz, Jaracza Street 63, 90-251 Lodz, Poland; ^4^Cardiac Rehabilitation Centre, Copernicus Memorial Hospital, Lodz, Popioly Street 40, 93-438 Lodz, Poland; ^5^Department of Physical Medicine, Medical University of Lodz, Pl. Hallera 1, 90-647 Lodz, Poland; ^6^Department of Clinical Physiology, Medical University of Lodz, Mazowiecka Street 6/8, 92-215 Lodz, Poland; ^7^Department of Sports Medicine, Medical University of Lodz, Pomorska Street 251, 92-213 Lodz, Poland

## Abstract

Whether the incidence of coronary heart disease (CHD) is related to a decrease in total antioxidant capacity (TAC) has not yet been completely clarified. We assessed TAC of blood serum in a group of 163 men with CHD aged 34.8–77.0 years and in 163 age-matched peers without CHD. Two spectrophotometric methods were applied to assess TAC: ferric reducing ability of serum (TAC-FRAS) and 2.2-diphenyl-1-picryl-hydrazyl (TAC-DPPH) tests. In the CHD group, multivariate analysis revealed that uric acid (UA), triglycerides, and systolic blood pressure contributed independently to the TAC-FRAS variance. TAC-DPPH was favorably predicted by UA concentration, but negatively so by current smoking and glucose levels. In men without CHD, UA was the only independent determinant of both TAC-FRAS and TAC-DPPH. Presence of CHD was not an independent predictor of TAC—observed between-group differences (higher TAC in CHD patients) disappeared after adjustment for other confounders. We conclude that UA is the main determinant of TAC of blood serum in men. TAC is not directly influenced by age or CHD but is related to several indices of overweight/obesity and laboratory measures of metabolic syndrome, especially in patients with CHD.

## 1. Introduction

An increasing number of studies focus on the role of reactive oxygen species (ROS) in the pathogenesis of premature ageing as well as of numerous civilization diseases, such as cardiovascular diseases [1–3]. It has been suggested that higher antioxidant potential can protect the organism against undesirable ROS activity and thus prevent disease incidence [1]. However, the present state of knowledge on such dependence is still not complete [4].

Coronary heart disease (CHD) is the most important cause of mortality in developed countries. Numerous discrepancies have been observed in the study results and no unequivocal answer has been reached whether the incidence of CHD is related to a decrease in antioxidant potential. Relationship of CHD to antioxidant defenses may be modified not only by many demographic, anthropometric, physiological, and biochemical confounders but also by different exogenic substances such as applied medications or cigarette smoking [5, 6].

Total antioxidant capacity (TAC) assessment is an established methodology to measure different elements of antioxidant defense system together [7]. In order to assess TAC several methods are available. The final value of measured TAC in the sample often depends on the procedure used in every specific assay. Ferric reducing ability of serum (FRAS) is an established TAC measuring test, being a modification of the ferric reducing ability of plasma (FRAP) [8] method commonly used for TAC measurement. Recently, a new spectrophotometric 2.2-diphenyl-1-picryl-hydrazyl (DPPH) test has also been proposed to measure TAC even more reliably [9].

Therefore, the aim of the present study was to compare TAC in CHD patients and in healthy age-matched subjects, taking into account anthropometric and biochemical correlates.

## 2. Methods

### 2.1. Subjects

The study was carried out in the two age-matched groups of men. Group I consisted of 163 CHD patients aged 34.8–77.0 (56.59 ± 8.04) years. In the course of myocardial ischemia and reperfusion the increased concentration of free radicals may also cause an increase in antioxidant enzymes activities. In order to exclude the possibility of acute ischemia-reperfusion reactions we qualified the patients in whom the most recent acute coronary event, cardiac or cardio-surgery intervention had occurred at least a minimum of one month earlier. Among the males with CHD 130 had a history of myocardial infarction (MI) (13 patients-twice), 137 underwent coronary catheterization, 107 underwent percutaneous transluminal coronary angioplasty (PTCA), 23 underwent coronary artery bypass surgery (CABG), 107 men demonstrated arterial hypertension (HA), and 25 displayed diabetes mellitus (DM). An applied pharmacotherapy regimen usually involved aspirin (*n* = 147), statins (*n* = 142), fibrates (*n* = 4), beta-blockers (*n* = 134), angiotensin-converting enzyme (ACE) inhibitors (*n* = 87), ticlopidine (*n* = 51), long-acting nitrates (*n* = 48), clopidogrel (*n* = 25), diuretics (*n* = 25), calcium channel blockers (*n* = 16), oral antidiabetic drugs (*n* = 18; sulfonylureas-11, metformin-9, acarbose-2), and insulin (*n* = 4).

To every patient, an age-matched peer without CHD was assigned. Control group consisted of men who attended the Healthy Men Centre of the Medical University of Lodz and were regularly monitored at least once a year. All the participants were relatively healthy community-dwelling men able and willing to visit the outpatient clinic as well as to take part in the multiple examinations. Group II comprised 163 males aged 34.3–76.1 (56.66 ± 7.99) years. Thirty-five of these men had HA and were treated with beta-blockers (*n* = 13) and ACE inhibitors (*n* = 24). Fourteen men were treated for hypercholesterolemia with statins and 16 used preventive treatment with low-dose aspirin. All the subjects in the study were free from known malignant diseases, important chronic inflammatory diseases, renal disorders, disability, or dementia. Apart from salt, glucose, and cholesterol limitations, none of the subjects was following a special diet. The presence of CHD was excluded in the control group based on clinical examination and exercise testing. The graded submaximal exercise test was carried out on a Monark type 818E (Stockholm, Sweden) bicycle ergometer with 30 watt increments every 3 min to achieve at least 85% of maximal age-predicted heart rate (220-age) with a continuous ECG tracing. All the subjects were informed of the purpose of the study. The study had been approved by the Ethics Committee and written informed consent was obtained from all the subjects.

### 2.2. Protocol and Measures

The subjects were asked to report to the Research Centre between 8.00 and 9.00 a.m. after overnight fasting for a minimum of 12 hours (they could consume a light supper without animal-derived fats the previous day, but no other particular dietary instructions were given) and after overnight rest, restraining from physical exercises, smoking, and alcohol for at least 12 h before laboratory measurements. After fasting blood drawing all the participants were given a light breakfast and a multidimensional assessment was performed with each subject. During the standard medical interview the applied pharmacotherapy regimen was assessed. The smoking and usual dietary habits were evaluated using the World Health Organization Countrywide Integrated Noncommunicable Diseases Intervention (WHO CINDI) questionnaire [10].

### 2.3. Anthropometric Data

Anthropometric data was collected by standard methods. Height and weight were measured and the body mass index-BMI (kg*·*m^−2^) was calculated. Skinfold measurements were taken at four sites: triceps, biceps, subscapula, and supraileum. The percentage of body fat was estimated from skinfold measurements according to Durnin and Womersley [11]. Measurements of waist and hip circumference were taken and waist-to-hip ratio (WHR) was calculated as an index of visceral obesity.

### 2.4. Laboratory Measurements

Fasting blood samples were drawn from the antecubital vein: for measurements of TAC into Vacuette tubes (Greiner Bio One GmbH, Kremsmunster, Austria) with sodium heparin (200 mg*·*L^−1^) or into siliconized tubes (for other tests). Enzymatic methods were used to determine serum total cholesterol (TC; CORMAY LiquickCor-CHOL), triglycerides (TG; CORMAY LiquickCor-TG), glucose (CORMAY LiquickCor-GLUCOSE), and uric acid concentrations (UA; CORMAY LiquickCor-UA). High density lipoprotein cholesterol (HDL-C) was measured by the precipitation method (CORMAY-HDL). Low density lipoprotein cholesterol (LDL-C) was estimated using the Friedewald formula.

### 2.5. Total Antioxidant Capacity

Blood samples were incubated for 30 minutes at 37°C and then centrifuged for 10 minutes (4°C, 1500 ×g) for further TAC measurements. Subsequently the samples were stored at −80°C for no longer than 30 days prior to the assays of antioxidant activity [9]. The measurements of blood serum TAC were performed using two spectrophotometric methods: the FRAS method (ferric reducing ability of serum) originally described by Benzie and Strain [8] with some modifications [9] and the DPPH method (2.2-diphenyl-1-picryl-hydrazyl) [9]. To get reliable data, all individual results were calculated as a mean from three separate measurements. Mean coefficients of variation across the triplicate measurements (*n* = 30) calculated for TAC-DPPH and TAC-FRAS were 0.049 and 0.018, respectively. In addition, both TAC assessments were performed in parallel using the same laboratory equipment (spectrophotometer) and within the same time frame. TAC-FRAS values are expressed in mmol*·*L^−1^ of formed FeCl_2_ (mmol FeCl_2_ *·*L^−1^). DPPH test consists of the scavenging of free radical DPPH (a relatively stable compound in alcoholic solution with a peak absorbance at *λ* = 517 nm) by a complex of antioxidants in the assayed sample of deproteinized serum. A decline of absorbance values equivalent to % of DPPH reduction expresses the level of the TAC-DPPH. The precise methodology of both tests has been described elsewhere [9].

### 2.6. Statistical Analysis

Data were verified for normality of distribution and equality of variances. Variables that did not meet the assumption of normality were analyzed with nonparametric statistics. A one-way analysis of variance (ANOVA) with Bonferroni* post hoc* testing, the Kruskal-Wallis test, and chi-square test (with Yates' correction for 2 × 2 tables) were used for comparison between the two groups of men. Pearson product moment or Spearman correlations were used to determine the relationships between variables. Multiple linear regression (with forward stepwise technique) was used with all the independent variables to select variables that independently predict TAC levels. Comorbidities, drugs, and current smoking were entered as dummy variables (yes = 1; no = 0). TAC-FRAS values were normalized using a log transformation and TAC-DPPH values were normalized using a square root transformation for the purpose of statistical analyses. The results are presented as the mean ± standard deviation. The level of significance was set at *P* ≤ 0.05 for all the analyses.

## 3. Results

Table 1 shows baseline characteristics of both groups. Men with CHD were characterized by higher values of BMI, WHR, and percentage of body fat in comparison with the men without CHD. CHD patients had lower values of TC, LDL-C, and HDL-C concentrations, lower TC/HDL-C ratio, and higher values of TG. CHD patients had higher values of both TAC-FRAS and TAC-DPPH (Table 1).

**Figure 1:**
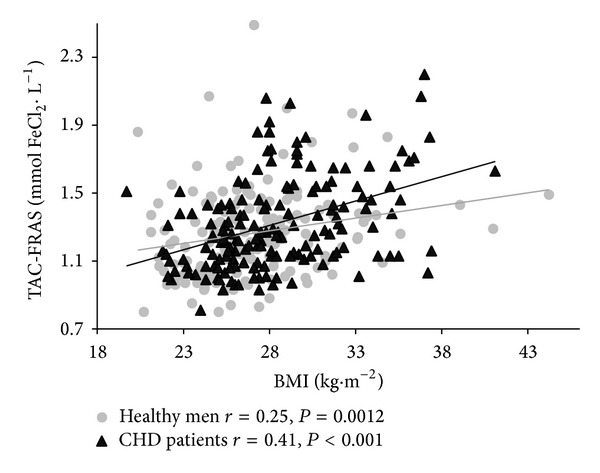
Correlation between TAC-FRAS and body mass index in men without CHD and in patients with CHD. TAC—total antioxidant capacity; FRAS—ferric reducing ability of serum; BMI—body mass index; CHD—coronary heart disease.

**Figure 2:**
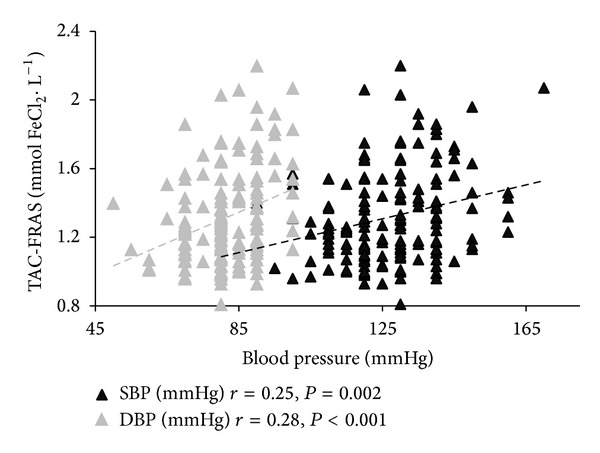
Correlation between TAC-FRAS and values of blood pressure in patients with coronary heart disease. TAC—total antioxidant capacity; FRAS—ferric reducing ability of serum; SBP—systolic blood pressure; DBP—diastolic blood pressure.

**Figure 3:**
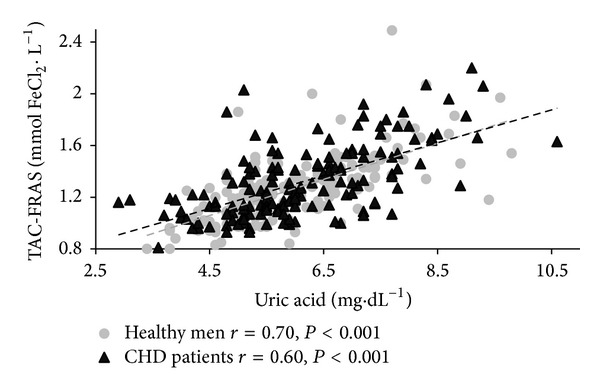
Correlation between TAC-FRAS and uric acid concentration in men without CHD and in patients with CHD. TAC—total antioxidant capacity; FRAS—ferric reducing ability of serum; CHD—coronary heart disease.

**Figure 4:**
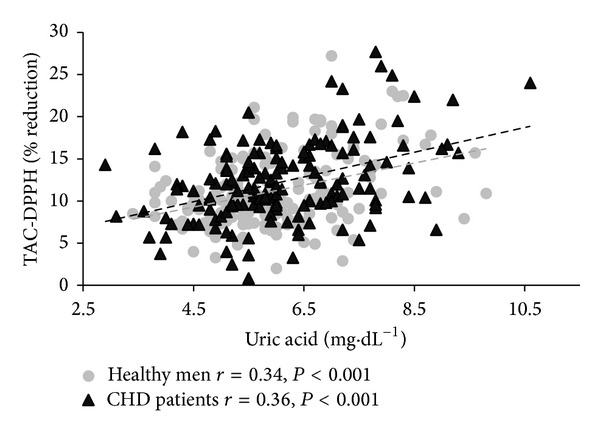
Correlation between TAC-DPPH and uric acid concentration in men without CHD and in patients with CHD. TAC—total antioxidant capacity; DPPH—2.2-diphenyl-1-picryl-hydrazyl; CHD—coronary heart disease.

Correlations between TAC, UA, and selected anthropometric, biochemical, and blood pressure characteristics for both groups are shown in Table 2. Age was not a determinant affecting the antioxidative barrier, regardless of the presence of CHD. In both groups TAC and UA were positively related to several anthropometric overweight/obesity measures (Figure 1). In CHD patients, SBP and DBP were positively correlated with TAC-FRAS (Figure 2) while DBP was positively correlated with UA. In the CHD group, higher values of TC and TG concentrations corresponded with higher values of TAC-FRAS, while TAC-DPPH was inversely related to glucose concentration. In both groups, TAC-FRAS and TAC-DPPH strongly positively correlated with UA (Figures 3 and 4). A positive intercorrelation was also found between TAC-FRAS and TAC-DPPH both in men without CHD (*r* = 0.38; *P* < 0.001) and in CHD group (*r* = 0.17; *P* = 0.03).

Currently nonsmoking cardiac patients (*n* = 140) were characterized with significantly higher (*P* < 0.01) values of TAC-FRAS (1.33 ± 0.27 mmol FeCl_2_ *·*L^−1^) and TAC-DPPH (13.0 ± 4.93% reduction) than ones who were currently smoking (1.20 ± 0.27 mmol FeCl_2_ *·*L^−1^ and 9.4 ± 3.56% reduction, resp.).

Higher values of TAC-DPPH were found in patients taking aspirin, beta-blockers, ACE inhibitors, ticlopidine, and clopidogrel in the CHD group. In individuals without CHD, TAC-FRAS was higher in males taking statins and beta-blockers.

### 3.1. Multivariate Analyses

All the comorbidities, cardiovascular risk factors assessed, and pharmacotherapy were included, together with age, smoking habit, and CHD presence, into multiple regression models. Continuous variables such as BMI or WHR were introduced unchanged to those models in order to retain precision which would be lost while converting those data to categorical variables (e.g., overweight/obesity categories).

In group I (CHD patients), TAC-FRAS was favorably predicted by UA, TG, and SBP:

(1) log⁡⁡TAC-FRAS=−0.589+0.0777×UA+0.000473×TG   +0.00234×SBP(adjusted  R2=44.8%).

TAC-DPPH was favorably predicted by UA while negatively so by glucose and current smoking. Consider

(2) squared  root  TAC-DPPH   =2.782+0.2038×UA−0.00532×glucose    −0.3965×current  smoking∗(adjusted  R2=23.8%),

*yes = 1; no = 0.

When current pharmacotherapy was taken into account, ticlopidine and clopidogrel contributed also to the TAC-DPPH variance [12].

In group II (men without CHD), both TAC values were predicted only by UA. Consider

(3) log⁡⁡TAC-FRAS=−  0.4311+0.1081 ×UA(adjusted  R2=47.4%)squared  root  TAC-DPPH=2.209+0.1806 ×UA(adjusted  R2=11.4%).

Presence of CHD was not an independent predictor of TAC—observed between-group differences for TAC disappeared after adjustment for other confounders. In the combined population of all 326 studied men, TAC—FRAS was favorably predicted by UA, TG, and DBP. Consider

(4) log⁡⁡TAC-FRAS=−0.559+0.0935×UA+0.00027×TG +0.00232×DBP(adjusted  R2=45.3%).

TAC-DPPH was favorably predicted by UA while negatively so by glucose and current smoking. Consider

(5) squared  root  TAC-DPPH =2.555+0.1992×UA−0.00368  ×glucose−0.2183×current  smoking∗  ×(adjusted  R2=16.7%),

*yes = 1; no = 0.

## 4. Discussion

Previous studies provided confusing results on the relationship of the presence of cardiovascular diseases to TAC. Several studies found significantly lower blood antioxidants and TAC in patients with CHD [13, 14]. Similarly, several studies found that in metabolic syndrome and HA patients exhibit decreased antioxidant protection and increased lipid peroxidation [15, 16]. However, no significant changes of TAC were observed during and after the incidence of MI [17] or between hypertensive patients and normal controls [18]. Vassalle et al. [19] identified higher values of TAC measured by OXY-adsorbent test in hypertensive individuals in comparison to subjects without HA. Elevated values of TAC were also found in patients with atherosclerosis in comparison with healthy age-gender-matched counterparts [20]. The analysis performed in the present study showed an increase of TAC in men with CHD or HA, but only in bivariate associations. After adjustment for common cardiometabolic risk factors, these changes were not detectable. Instead, positive correlations of TAC with indices of overweight/obesity in both groups and positive relationships to blood pressure and laboratory lipid indices of metabolic syndrome in CHD patients were found. One possible explanation of these findings may be that in the course of many diseases, lower TAC may be due to the depletion of the antioxidant barrier as an effect of long term oxidative stress [21, 22]. In early stages of those disorders, but already in the presence of risk factors (overweight/obesity, elevated blood pressure, and laboratory lipid indices of metabolic syndrome), the antioxidant defense system may respond to sustained oxidative stress by increasing its activity. Similar observations have been reported in some earlier studies [19, 23, 24]. For example, Vassalle et al. [19] observed higher TAC in patients with hypertension while in several studies a positive relationship between antioxidant potential and BMI was found [23, 24]. On the other hand, an inverse correlation between dietary TAC and BMI in obese children and adolescents [25] and between dietary TAC and central adiposity measurements in healthy young adults [26] was found. Plasma TAC values were also not different between pre- and postoperative morbid obesity patients, as well as versus controls [27].

In our study, several direct correlations for obesity measures and TAC were observed both in healthy men and in men with CHD. These associations were more visible for TAC-FRAS and in CHD patients. In CHD patients several significant positive correlations were also observed between TAC-FRAS and other cardiovascular risk factors as elevated TC, TG, and blood pressure. The explanation of these associations may be based on the classical physiological concept of hormesis. This Greek word means a state in which a sublethal dose of toxin can increase the tolerance of organism to stand up to higher doses of toxin [28]. In obese subjects, higher levels of derivatives of ROS metabolites were found [29]. Thus, the best strategy to enhance endogenous antioxidant level may actually be the oxidative stress itself [30]. It means that overweight/obesity may stimulate TAC and high values of antioxidant potential among obese individuals may be explained by secondary response to intensified oxidative stress characterizing subjects with higher amount of adipose tissue [31–33]. Therefore, these two phenomena should not be separated but rather seen as a sequence of incidents.

The next discrepancy appears in correlation with TAC and age. Our findings are contrary to several other studies which report that antioxidant capacities of human blood serum decrease along with age [34, 35] and the systemic oxidative stress status is higher in older subjects [36]. Comparison between middle-aged and older subjects suggested a progressive and slow decline of antioxidant status [37]. On the other hand, comparison of active elderly group with active younger one did not show the difference in the level of antioxidant defence and in the level of peroxidation [38]. Interestingly, in the study involving 2828 subjects from the Framingham Heart Study age was inversely related to urinary creatinine-indexed 8-epi-PGF2alpha levels (marker of systemic oxidative stress) [32]. Some data showed that TAC did not differ between healthy elderly men and young ones, but some individual indices of antioxidant defence system were even higher in the elderly, for instance, red blood cell and plasma GSH-Px [39]. In our study, there was no statistically significant relation between age and blood serum TAC in men either with or without CHD. These results may suggest that along with age the activity or the amount of single antioxidants may alter but TAC remains essentially unchanged.

Depletion of the antioxidant barrier may occur during prolonged exposure to sustained oxidative stress, with this being the case with current smoking and probably also elevated glucose levels in our CHD patients. It has been well documented that cigarette smoke increases the level of oxidative stress, decreases antioxidant capacities, and induces several chronic diseases (especially CHD, HA, MI, and stroke) [40]. It has been found that acute and chronic cigarette smoking impairs the relaxation of large blood vessels mediated by nitric oxide synthase. Cigarette smoke extract is known to contain a considerable amount of ROS such as superoxide anions, hydroxyl radicals, or hydrogen peroxides [41]. Our results are in agreement with these investigations. In the present study currently smoking subjects with CHD demonstrated lowered antioxidant capacities of blood serum. These relations were less visible in individuals without CHD, suggesting that in CHD patients, the antioxidant defense system is more vulnerable to permanent oxidative stress connected with cigarette smoke.

UA, which also serves as a water-soluble antioxidant and a free radical scavenger in humans, is an end-product of purine metabolism and is present in high concentrations in plasma [20, 42]. Systemic administration of UA causes an increase in serum antioxidant capacity [43]. There is some evidence that increased oxidative stress is associated with higher level of circulating UA and that it may protect against oxidative modification of endothelial enzymes and preserve the ability of the endothelium to mediate vascular dilatation in oxidative stress [42, 44, 45]. Hyperuricemia may be a compensatory mechanism to counteract oxidative damage related to atherosclerosis and ageing in humans [20]. On the other hand, its high concentration is one of the risk factors of CHD [5]. Our data bring some interesting information, as uric acid levels were not different in the two groups and were not related to age in any of the studied groups. UA was found to be the strongest predictor of antioxidant status, irrespective of TAC assay and studied group. Similar predominant effect on antioxidant potential has also been noted in other studies [46]. Undoubtedly, this two-directional activity of uric acid, as both potent antioxidant and cardiovascular risk factor, would require further studies [45–47].

Several shortcomings of the present study should be acknowledged. This is a cross-sectional study performed in volunteers: CHD patients and their age-matched peers. Active well-educated subjects in relatively good health are more prone to participate in such studies. Antioxidants status is the result of the interaction of many different compounds and systemic metabolic connections and the TAC is modulated either by ROS overload (not measured in the present study) or by antioxidants concentration. Correlations between TAC and selected patients characteristics were relatively modest. Although both DPPH and FRAS tests measure the TAC of blood serum, they reflect somewhat different physiological properties [9]. Future studies, taking into account also oxidative and inflammatory stress indices, are needed to confirm these associations. Future studies should also relate compensatory antioxidative response to the duration of distinct diseases and disorders.

We conclude that TAC, assayed with either the FRAS or the DPPH methods, does not differ between men with and without CHD when additional confounders are taken into consideration. Age was not a determinant affecting the antioxidative barrier, regardless of the presence of CHD. In both groups, UA was the strongest determinant of TAC-FRAS and TAC-DPPH. TAC is directly related to several indices of overweight/obesity and laboratory measures of metabolic syndrome, especially in patients with CHD.

## Figures and Tables

**Table 1:** Age, selected anthropometric and biochemical characteristics, cigarette smoking, blood pressure, and total antioxidant capacity in men with CHD and in their peers without CHD.

Variable	Group I (CHD patients) *n* = 163	Group II (without CHD) *n* = 163
Age (years)	56.59 ± 8.04	56.66 ± 7.99
Body mass (kg)	85.4 ± 13.5	83.0 ± 13.1
BMI (kg*·*m^−2^)	28.4 ± 3.9^†^	27.1 ± 3.8
Waist circumference (cm)	101.8 ± 9.6^‡^	94.9 ± 10.1
WHR	0.98 ± 0.05^‡^	0.94 ± 0.06
Percentage of body fat	24.9 ± 5.3*	23.6 ± 5.8
Past smokers (% of *n*)	83.9^‡^	56.4
Current smokers (% of *n*)	13	13
SBP (mmHg)	127.3 ± 14.7	127.8 ± 13.9
DBP (mmHg)	81.9 ± 9.1	82.6 ± 9.3
TC (mg*·*dL^−1^)	179.1 ± 42.7^‡^	217.1 ± 43.2
LDL-C (mg*·*dL^−1^)	104.3 ± 37.1^‡^	142.6 ± 40.6
HDL-C (mg*·*dL^−1^)	45.1 ± 9.5^‡^	50.7 ± 11.6
TC/HDL-C ratio	4.05 ± 1.1^†^	4.48 ± 1.3
TG (mg*·*dL^−1^)	150.1 ± 91.3^‡^	125.6 ± 85.2
Glucose (mg*·*dL^−1^)	100.1 ± 30.1	99.3 ± 21.1
UA (mg*·*dL^−1^)	6.13 ± 1.35	5.98 ± 1.23
TAC-FRAS (mmol FeCl_2_ *·*L^−1^)	1.32 ± 0.27*	1.26 ± 0.26
TAC-DPPH (% reduction)	12.5 ± 4.9*	11.2 ± 4.4

*P < 0.05, ^†^ *P* < 0.01, and ^‡^ *P* < 0.001 as compared to the healthy subjects.

BMI: body mass index; WHR: waist-to-hip ratio; SBP: systolic blood pressure; DBP: diastolic blood pressure; TC: total cholesterol; LDL-C: low density lipoprotein cholesterol; HDL-C: high density lipoprotein cholesterol; TG: triglycerides; UA: uric acid; TAC: total antioxidant capacity; FRAS: ferric reducing ability of serum; DPPH: 2.2-diphenyl-1-picryl-hydrazyl; CHD: coronary heart disease.

**Table 2:** Correlation coefficients of total antioxidant capacity measures and uric acid concentration to age, selected anthropometric, biochemical, and blood pressure characteristics in men with and without CHD.

Variable	Group I (CHD patients)			Group II (without CHD)		
	TAC-FRAS (mmol FeCl_2_ *·*L^−1^)	TAC-DPPH (% reduction)	UA (mg*·*dL^−1^)	TAC-FRAS (mmol FeCl_2_ *·*L^−1^)	TAC-DPPH (% reduction)	UA (mg*·*dL^−1^)
Age (years)	−0.09	−0.06	−0.08	0.08	0.006	0.01
Body mass (kg)	0.33^‡^	0.05	0.28^‡^	0.20^†^	−0.06	0.30^‡^
BMI (kg*·*m^−2^)	0.41^‡^	0.04	0.28^‡^	0.25^†^	0.02	0.33^‡^
Waist circumference (cm)	0.32^‡^	0.16*	0.25^†^	0.21^†^	−0.02	0.35^‡^
WHR	0.22^†^	0.23^†^	0.18*	0.22^†^	0.13	0.33^‡^
Percentage of body fat	0.30^‡^	−0.02	0.16*	0.11	0.07	0.26^†^
SBP (mmHg)	0.25^†^	0.01	0.10	0.09	0.06	0.05
DBP (mmHg)	0.28^‡^	0.02	0.18*	0.14	0.09	0.06
TC (mg*·*dL^−1^)	0.16*	−0.06	0.08	−0.01	−0.11	−0.07
LDL-C (mg*·*dL^−1^)	0.04	−0.10	−0.07	−0.03	−0.11	−0.07
HDL-C (mg*·*dL^−1^)	−0.05	−0.04	−0.04	−0.05	−0.09	−0.11
TC/HDL-C ratio	0.12	−0.03	0.05	0.03	0.02	0.04
TG (mg*·*dL^−1^)	0.24^†^	0.07	0.31^‡^	0.12	−0.01	0.13
Glucose (mg*·*dL^−1^)	0.14	−0.25^†^	0.09	0.06	0.02	0.09
UA (mg*·*dL^−1^)	0.60^‡^	0.36^‡^		0.70^‡^	0.34^‡^	

*P < 0.05, ^†^ *P* ≤ 0.01, ^‡^ *P* < 0.001.

BMI: body mass index; WHR: waist-to-hip ratio; SBP: systolic blood pressure; DBP: diastolic blood pressure; TC: total cholesterol; LDL-C: low density lipoprotein cholesterol; HDL-C: high density lipoprotein cholesterol; TG: triglycerides; UA: uric acid; TAC: total antioxidant capacity; FRAS: ferric reducing ability of serum; DPPH: 2.2-diphenyl-1-picryl-hydrazyl; CHD: coronary heart disease.
